# Digital health literacy and use of patient portals among Spanish-preferred patients in the United States: a cross-sectional assessment

**DOI:** 10.3389/fpubh.2024.1455395

**Published:** 2024-12-10

**Authors:** Lindsey M. Philpot, Priya Ramar, Daniel L. Roellinger, Margaret A. McIntee, Jon O. Ebbert

**Affiliations:** ^1^Division of Community Internal Medicine, Geriatrics, and Palliative Care, Department of Medicine, Mayo Clinic, Rochester, MN, United States; ^2^Division of Epidemiology, Department of Quantitative Health Sciences, Mayo Clinic, Rochester, MN, United States; ^3^Administrative Services, Mayo Clinic, Rochester, MN, United States

**Keywords:** patient portals, digital health, Technology Acceptance Model, digital health literacy, Limited English Proficiency, Spanish preferred

## Abstract

**Objective:**

Individuals with Limited English Proficiency (LEP), including Spanish-preferred patients, face healthcare challenges due to language barriers. Despite the potential of digital health technologies to improve access and outcomes, there is a “digital divide” with underutilization among vulnerable populations, including Spanish-speaking LEP individuals, highlighting a need for increased understanding and equitable digital health solutions.

**Materials and methods:**

A multi-mode, multi-language cross-sectional survey was built based on the Technology Acceptance Model and deployed from a multi-state healthcare practice. Measures included patient-reported comfort level with reading and speaking English, internet and computer access and satisfaction, ability to perform healthcare-related online tasks, and the eHEALS scale of digital health literacy.

**Results:**

A total of 212 Spanish-preferred patients completed the survey (response rate, 212/2,726 = 7.8%), of which 73.6% indicated lack of comfort in reading or writing in English (LEP *n* = 156). Spanish-speaking individuals with LEP reported higher rates of needing help when learning how to use new technology or devices, reporting difficulty in the evaluation of health information on the internet and being able to differentiate high-quality information from low-quality online health resources, feeling confident in using health information found online to make health decisions, and having lower access to health-related online services than Spanish-speaking individuals without LEP.

**Discussion:**

Improving equitable accessibility to digital tools for individuals with LEP seeking healthcare can help to improve their engagement with their providers and promote self-efficacy in their care. Opportunities exist with emerging technologies to develop language-concordant healthcare resources that will improve outcomes for Spanish-preferred patients.

## Introduction

According to the U.S. Census Bureau, 67.3 million residents of the United States (US) spoke a language other than English at home in 2018 (1). Spanish speakers comprise the second largest spoken language group within the US. In 2020, approximately 15.9 million individuals in the U.S. primarily spoke Spanish and indicated limited ability to understand spoken or written English (2). Individuals with Limited English Proficiency (LEP) are those who have limited ability to read, speak, or understand English (3). Most individuals with LEP indicate that English is not their first spoken language, or the primary language spoken within their home (4).

Individuals in the United States with LEP have unique challenges related to health and healthcare. As lack of language concordance impacts basic communication tasks, patients with LEP experience miscommunication that leads to misdiagnosis, incorrect or delayed medical care, and exclusion from health-related programs and services from which they could benefit most (4). They have longer hospital stays when professional interpreters are not included within the hospital admission or discharge processes, and have higher rates of readmissions for chronic health conditions due to communication gaps related to health management and medications (4). Concerns over equitable access to care have led to the creation of federal mandates that patients with LEP hold legal rights to access healthcare in their preferred language (5). The U.S. Department of Health and Human Services created the Language Access Plan to help facilitate greater access to healthcare services for individuals with LEP (3). Digital health, or technology-enabled tools intended to improve health and healthcare, may have a role to play in mitigating experiences of inequity to improve healthcare access and health outcomes. Among populations experiencing health disparities, digital health facilitates recruitment and engagement in clinical research through enabling a wider reach to populations not traditionally seeking care from academic medical centers and enabling protocols that are more accessible and convenient to research participants (6, 7) creates more direct access to experts in health and healthcare as medical specialists are often concentrated at tertiary and academic medical centers (8) improves access to healthcare services despite geographic location and time of day, such as patients residing in rural or urban areas with decreased access to specialist-level care (9) and customizes interventions based on cultural characteristics of individuals and communities, such as through language translation and reading level (10).

Despite the hope and evidence that digital health technologies will facilitate equitable access to health and healthcare, trends in digital health use among vulnerable populations, including those with LEP and those who recently immigrated to the US, do not demonstrate increased utilization (11). Research to date shows that Spanish-preferred individuals in the US experience language barriers associated with currently available digital health tools that are predominantly available only in the English language, and were less likely to utilize telehealth services during the COVID-19 pandemic compared to other ethnic groups, exemplifying the gap in digital health usability and use among this population (12). Digital equity is only achieved when every individual and community can fully participate in society, including health, well-being, and healthcare services. Inequitable access or ability to use digital health technologies by subgroups or communities of individuals is of concern and has been termed the “digital divide” (13). As digital equity is a relatively new concept, substantial gaps in our knowledge exist regarding the use of digital health to support health and healthcare among certain at-risk populations, including Spanish-speaking individuals with LEP in an English-predominant society like the US (14, 15).

## Materials and methods

### Study overview

**Figure 1:**
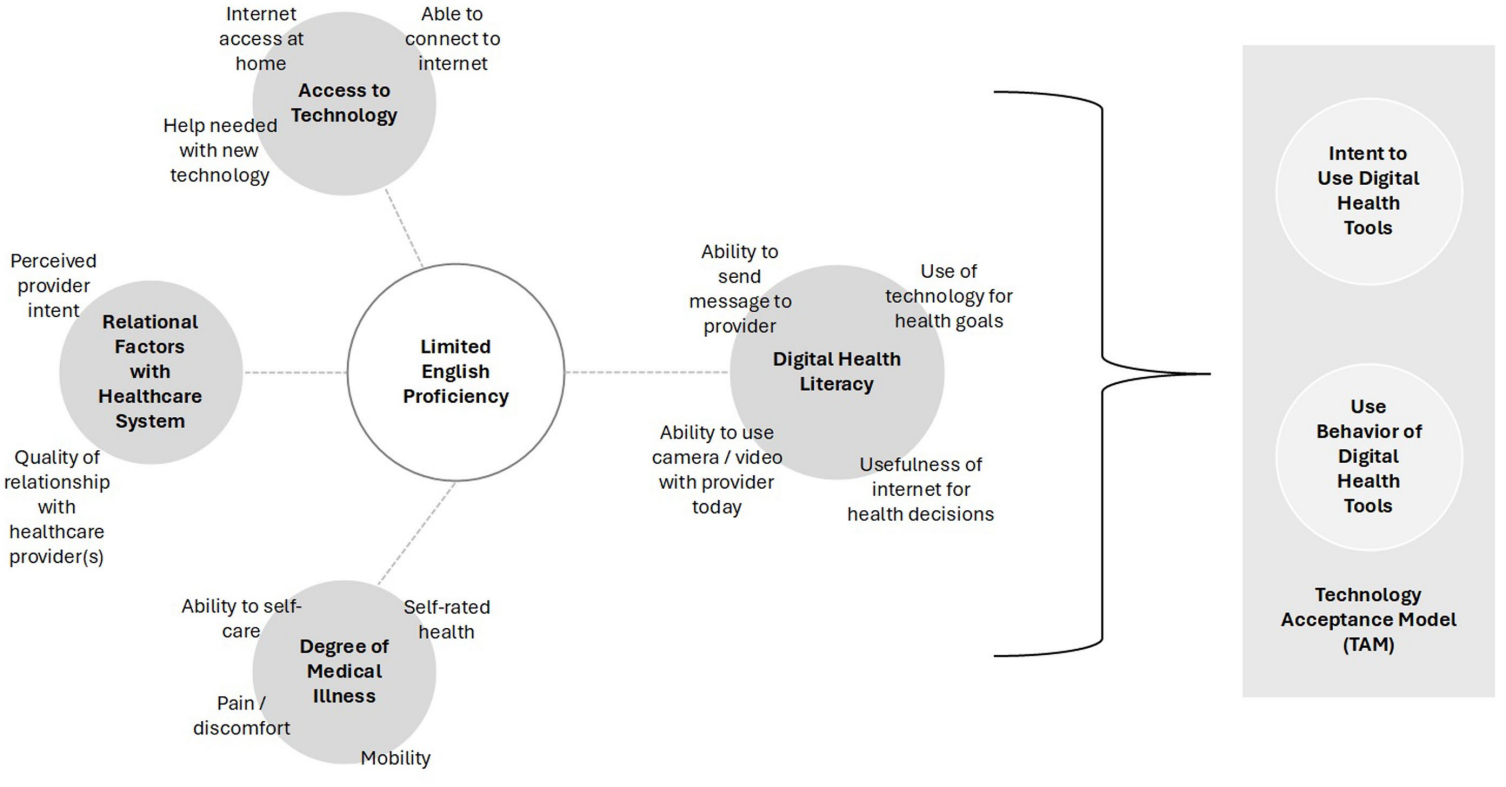
Theoretical model under investigation to determine how Limited English Proficiency (LEP) among Spanish-preferred patients is associated with access to technology, relational factors with the healthcare system, degree of medical illness, and digital health literacy.

In order to advance our understanding of the digital health use characteristics of Spanish-speakers with LEP, we performed a sub-analysis of a cross-sectional survey based on the domains of the Technology Acceptance Model (Figure 1) (16). The goal of the original investigation was to understand digital determinants of health across a general sample of patients receiving care at a multi-site, multi-state healthcare system, with an over-sampling of individuals with an indicated spoken language of Spanish. We developed and deployed a cross-sectional survey via two modes: an electronic survey delivered to patient-provided e-mail addresses and a paper survey delivered via U.S. mail to patient-provided addresses of permanent residence. Deployment mode was selected based on patient-provided preference for communications from our healthcare system. Electronic surveys were designed, managed, and deployed using Qualtrics Survey Software (Provo, UT) and included three total invitations to complete the survey instrument. Paper surveys were designed using InDesign Software and were deployed in a scannable booklet format in a single distribution wave. Stamped return envelopes were included with all mailed, paper surveys. Those who did not respond to their electronic survey after three electronic contacts received a paper survey. Responses to electronic and paper surveys were appended into a single data set for analysis purposes. The build, deployment, and management of survey instruments were performed by the Mayo Clinic Survey Research Center. The Mayo Clinic Institutional Review Board reviewed and approved this study as exempt (IRB # 22–008356; Principal Investigator: LM Philpot). Development and deployment of the survey instrument has been described previously (16). A copy of the survey instrument deployed is included as a Supplementary material.

### Study population

As part of the standard of care delivered to all patients seen at any Mayo Clinic site, patients are encouraged to register for an Epic patient portal that allows them to access their health information, upcoming and past medical appointments, and the ability to message with their care team, among other features. Since our deployment of the Epic electronic health record in 2018, over 1 million patients have received an invitation to register for an online patient portal account, with nearly 90% of patients choosing to enroll. The remaining 10% of patient portal accounts have registered but are inactive due to infrequent recent use, are pending activation by the end-user, or declined enrollment. Previous studies have reported that non-adopters of patient portal and digital health solutions respond to inquiries from healthcare provider initiative surveys at one-half the rate of those who do participate in these services. We oversampled at a 1: 2 ratio of active patient portal users to patients who declined to enroll, after limiting to patients who have had at least one healthcare interaction with Mayo Clinic in the 12 months prior to sampling. Patients were sampled if they were older than 18 years of age, not listed in the “do not contact” list, still living according to our internal records, and selected English or Spanish as their primary language within their medical record. Based on an anticipated response rate among patient portal users (20% response to electronic surveys, 15% response to paper surveys) and one-half the response rate among non-adopters (10% response to electronic surveys, 7.5% to paper surveys), we sampled approximately 3,000 active Epic patient portal users and 6,000 non-enrollees to allow approximately 1,000 in each class for analyses. The present study focused on Spanish-speaking respondents.

### Measures

Limited English Proficiency (LEP) was defined by a negative response to being comfortable reading or speaking English while “Non LEP” was defined by a positive response to being comfortable reading or speaking English.

Computer and Internet Use was defined by a positive response to ever connecting to the internet to surf the web or send and receive emails, accessing the internet from home, satisfaction with ability to access the internet, being the first among peers to try out new technology, and not needing help to set up a new electronic device.

Health and Healthcare-related use of electronic devices was defined by a positive response to being able to easily send a message to doctor today, being able to easily see a doctor using camera or video, use a tablet or smartphone to achieve a health-related goal, usefulness of the internet in making health-related decisions, and importance of accessing health resources on the internet.

Digital health literacy were measured independently as defined by Norman et al. (17) and included positive responses to knowing what health resources are available on the internet, where/ how to find, evaluate and use health resources on the internet, how to use the internet to answer questions about health, distinguishing high-quality health resources from low-quality health resources on the internet, and feeling confident in using information from the internet to make health decisions (14).

We also included survey item measures for patient demographics [i.e., age, gender, race, medical facilitators/barriers measured using EuroQol 5-dimension (EQ-5D) index] (15) and relational facilitators/barriers as measured by social support for patient portal use and relationship with healthcare provider(s). Race was defined based on patient feedback on the initial survey instrument during face validation of our survey instruments, which had originally proposed definitions used by the US Census Bureau (25). The final survey instrument, drafted in English, underwent translation into Spanish with linguistic validation by a third-party translation service provider (Morningside Translations, LLC). Deployed survey instruments are included as Supplementary material 1 (English-language version) and Supplementary material 2 (Spanish-language version).

### Statistical analysis

Survey responses were aggregated across modalities. Analysis of differences in nonresponse was assessed by age, race, and gender. Responders who completed at least 90% of survey questions were included in analyses. Distributions of patient characteristics and technology-related measures were described between LEP and Non LEP groups. Continuous variables were described using mean and standard deviation or median and interquartile range in the case of skewed distribution. Categorical variables were described using frequencies and percents. Comparisons by LEP status were performed using Chi Square test for categorical variables, and either t-tests with pooled estimates for equal variances (age) or Wilcoxon two-sample test for continuous variables with a non-normal distribution (EQ-5D index). All analyses were conducted using Statistical Analysis Software (SAS) version 9.4 (Cary, North Carolina), and differences are considered significant when *p* < 0.05.

## Results

### Characteristics of Spanish-speaking response population by Limited English Proficiency (LEP) status

A total of 2,726 surveys were sent to patients who indicated in their patient record that Spanish is their preferred language for communication. Of those, 212 individuals responded to our survey (response rate = 212/2,726, 7.8%) (Table 1). Forty-seven percent (*n* = 100/212, 47.2%) of respondents completed the paper-based survey and 52.8% (*n* = 112/212) of respondents completed the electronic survey. Survey respondents had an average age of 60 years, predominately identified themselves as female, Mexican (*n* = 83/212, 39.7%) or Central/South American (*n* = 46/212, 22.0%) racial groups, reported “Good” or better health, and reported good relationships with their healthcare providers (*n* = 188/212, 89.5%). When grouped by LEP status, we observed significant differences in self-rated health (Chi Square = 17.08, *p* = 0.002).

**Table 1:** Characteristics of response population for patients indicating Spanish as their preferred language by Limited English Proficiency (LEP) status.

	Non LEP (*N* = 56)	LEP (*N* = 156)	Total (*N* = 212)	*p*-value
Age, years				0.972
Missing	0	0	0	
Mean (SD)	59 (15)	60 (15)	60 (15)	
Range	30–89	21–97	21–97	
Gender, *n* (%)				0.691
Missing	2	4	6	
Female	28 (51.9%)	81 (53.3%)	109 (52.9%)	
Male	24 (44.4%)	63 (41.4%)	87 (42.2%)	
Prefer not to answer	1 (1.9%)	7 (4.6%)	8 (3.9%)	
Other	1 (1.9%)	1 (0.7%)	2 (1.0%)	
Race, *n* (%)				0.289
Missing	2	1	3	
Black/African	1 (1.9%)	1 (0.6%)	2 (1.0%)	
Central/South American	14 (25.9%)	32 (20.6%)	46 (22.0%)	
Mexican	16 (29.6%)	67 (43.2%)	83 (39.7%)	
Mixed	6 (11.1%)	9 (5.8%)	15 (7.2%)	
Prefer not to answer	1 (1.9%)	10 (6.5%)	11 (5.3%)	
None/other	11 (20.4%)	28 (18.1%)	39 (18.7%)	
White	5 (9.3%)	8 (5.2%)	13 (6.2%)	
EuroQol 5-dimension (EQ-5D) Index				0.125
Missing	1	6	7	
Median (IQR)	0.9 (0.7, 1.0)	0.9 (0.7, 1.0)	0.9 (0.7, 1.0)	
In general, would you say your health is, *n* (%)				0.002
Missing	2	1	3	
Excellent	7 (13.0%)	13 (8.4%)	20 (9.6%)	
Very Good	21 (38.9%)	24 (15.5%)	45 (21.5%)	
Good	11 (20.4%)	65 (41.9%)	76 (36.4%)	
Fair	11 (20.4%)	43 (27.7%)	54 (25.8%)	
Poor	4 (7.4%)	10 (6.5%)	14 (6.7%)	
Good relationship with healthcare providers, *n* (%)				0.615
Missing	2	0	2	
Agree	49 (90.7%)	139 (89.1%)	188 (89.5%)	
Neither	4 (7.4%)	16 (10.3%)	20 (9.5%)	
Disagree	1 (1.9%)	1 (0.6%)	2 (1.0%)	
Healthcare providers have your best interest, *n* (%)				0.589
Missing	1	0	1	
Agree	51 (92.7%)	143 (91.7%)	194 (91.9%)	
Neither agree or disagree	2 (3.6%)	10 (6.4%)	12 (5.7%)	
Disagree	2 (3.6%)	3 (1.9%)	5 (2.4%)	

IQR, Interquartile range; SD, Standard deviation.

### General computer and internet use of Spanish-speaking response population by LEP status

Most respondents indicated that they connect to the internet for surfing the web or to use Email (*n* = 176/212, 84.6%), with those with LEP reporting lower use than those without LEP (Table 2). Most respondents reported having the ability to access the internet from home (*n* = 191/212, 91.4%), with 79.9% being satisfied with their ability to access the internet when needed (*n* = 159/212). When grouped by LEP status, there were no significant differences by reported ability to connect to the internet, ability to access the internet from home, satisfaction with ability to access the internet, and being the first among peers to try new technologies. Those with LEP had a greater proportion reporting a need for help when using a new electronic device (Non LEP n = 20/56, 37.7% vs. LEP n = 107/156, 69.5%, Chi Square = 18.02, *p* < 0.001).

**Table 2:** General computer and internet use of Spanish-speaking response population by Limited English Proficiency (LEP) status.

	Non LEP (*N* = 56)	LEP (*N* = 156)	Total (*N* = 212)	*p*-value
Connect to internet to surf web/email?				0.236
Missing	2	2	4	
Yes	49 (90.7%)	127 (82.5%)	176 (84.6%)	
No	3 (5.6%)	22 (14.3%)	25 (12.0%)	
I do not know	2 (3.7%)	5 (3.2%)	7 (3.4%)	
Access internet from home?				0.176
Missing	1	2	3	
Yes	51 (92.7%)	140 (90.9%)	191 (91.4%)	
No	3 (5.5%)	14 (9.1%)	17 (8.1%)	
I do not know	1 (1.8%)	0 (0.0%)	1 (0.5%)	
Satisfaction in ability to access internet?				0.249
Missing	4	9	13	
Satisfied	42 (80.8%)	117 (79.6%)	159 (79.9%)	
Neither	10 (19.2%)	23 (15.6%)	33 (16.6%)	
Dissatisfied	0 (0.0%)	7 (4.8%)	7 (3.5%)	
Need help using new electronic device?				0.001
Missing	3	2	5	
Yes	20 (37.7%)	107 (69.5%)	127 (61.4%)	
No	32 (60.4%)	43 (27.9%)	75 (36.2%)	
I do not know	1 (1.9%)	4 (2.6%)	5 (2.4%)	
First among peers to try new technologies?				0.545
Missing	3	2	5	
Yes	12 (22.6%)	26 (16.9%)	38 (18.4%)	
No	37 (69.8%)	111 (72.1%)	148 (71.5%)	
I do not know	4 (7.5%)	17 (11.0%)	21 (10.1%)	

### Health and healthcare-related use of electronic devices of Spanish-speaking response population by LEP status

Most respondents indicated the ability to easily send an electronic message to their healthcare provider today (*n* = 152/212, 73.1%), with significant differences by LEP status (Non LEP *n* = 48/56, 88.9% vs. LEP *n* = 104/156, 67.5%; Chi Square = 9.34, *p* < 0.001) (Table 3). We observed significance among our response groups regarding the ability to easily use a camera or video camera with a doctor today (Non LEP *n* = 47/56, 87.0%vs. *n* = 91/156, LEP 59.1%; Chi Square = 15.1, *p* < 0.001).Fewer than one-half of our respondents indicated that they have used a smartphone or tablet for a health-related goal (*n* = 86/212, 42.2%; Non LEP *n* = 30/56, 55.6% vs. LEP *n* = 56/156, 37.3%; Chi Square = 7.48, *p* = 0.024) Most respondents indicated that the internet is useful for health decision making and that the internet is important for health related resources.

**Table 3:** Health and healthcare-related use of electronic devices of Spanish-speaking response population by Limited English Proficiency (LEP) status.

	Non LEP (*N* = 56)	LEP (*N* = 156)	Total (*N* = 212)	*p*-value
Easily able to send a message to doctor today?				<0.001
Missing	2	2	4	
Yes	48 (88.9%)	104 (67.5%)	152 (73.1%)	
No	3 (5.6%)	29 (18.8%)	32 (15.4%)	
I do not know	3 (5.6%)	21 (13.6%)	24 (11.5%)	
Easily able to use camera/video with doctor today?				<0.001
Missing	2	2	4	
Yes	47 (87.0%)	91 (59.1%)	138 (66.3%)	
No	6 (11.1%)	35 (22.7%)	41 (19.7%)	
I do not know	1 (1.9%)	28 (18.2%)	29 (13.9%)	
Used a smartphone or tablet for health goal?				0.024
Missing	2	6	8	
Yes	30 (55.6%)	56 (37.3%)	86 (42.2%)	
No	24 (44.4%)	85 (56.7%)	109 (53.4%)	
I do not know	0 (0.0%)	9 (6.0%)	9 (4.4%)	
How useful is the internet for health decisions?				0.291
Missing	2	3	5	
Useful	41 (75.9%)	106 (69.3%)	147 (71.0%)	
Not useful	8 (14.8%)	19 (12.4%)	27 (13.0%)	
Unsure	5 (9.3%)	28 (18.3%)	33 (15.9%)	
How important is internet for health resources?				0.060
Missing	1	2	3	
Important	47 (85.5%)	108 (70.1%)	155 (74.2%)	
Not important	5 (9.1%)	20 (13.0%)	25 (12.0%)	
Unsure	3 (5.5%)	26 (16.9%)	29 (13.9%)	

### Digital health literacy by LEP status

**Figure 2:**
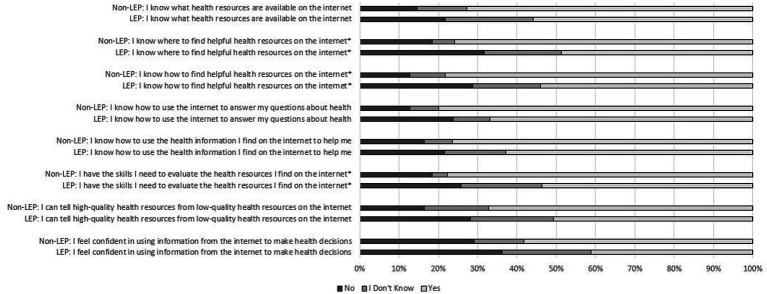
Digital health literacy of survey respondents by Limited English Proficiency (LEP) among Spanish-speaking patients within an English-dominant country.

Most patients, despite LEP status, indicated knowing what health resources are available (“I know what health resources are available on the internet.”), where to find help health resources (“I know where to find health resources on the internet.”), how to find helpful health resources (“I know how to find helpful health resources on the internet”), how to answer health-related questions (“I know how to use the internet to answer my questions about health”), and how to use health information found to help them (“I know how to use the health information I find on the internet to help me”) (Figure 2). We observed significantly different response distributions between those with LEP and those without LEP related to knowing where to find helpful health resources (“I know where to find help health resources on the internet”; Chi Square = 12.71, *p* < 0.001), knowing how to find helpful health resources (“I know how to find helpful health resources on the internet”; Chi Square = 9.89, *p* = 0.007), and feeling as though the respondent has the skills needed to evaluate health resources (“I have the skills I need to evaluate the health resources on the internet to help me”; Chi Square = 11.60, *p* = 0.003). We did not observe significant differences in distribution by LEP status among the remaining digital health literacy questions.

## Discussion

In this study, we observed that Spanish-speaking individuals who experience LEP report higher rates of needing help when learning how to use new technology or devices, reporting difficulty in the evaluation of health information on the internet and being able to differentiate high-quality information from low-quality online health resources, feeling confident in using health information found online to make health decisions, and having lower access to health-related online services than Spanish-speaking individuals without LEP.

Our investigation suggests individuals with LEP are experiencing access barriers to digital tools and technologies to support health and healthcare, which may explain their observed lower rates of telehealth service adoption (18). However, this lower rate of adoption may not be due to lack of interest, as a qualitative study of patient use of electronically-collected patient-reported outcome measures showed that individuals with LEP were interested in the use of digital tools to manage their care, including electronic patient reported outcome measures, while individuals without LEP indicated less interest (19). Language discordance may be another significant barrier to engagement with digital tools. Language-concordant care has generally been observed to be associated with better healthcare outcomes (20). In a secondary analysis of Kidney Awareness Registry and Education (KARE) pilot trial, patients with LEP were equally or more engaged with language-concordant, culturally appropriate telehealth interventions compared with their English-speaking counterparts (21). Adaptation of digital tools and technologies to make them language-concordant, facilitated by leveraging large language models (22), may enhance engagement among patients with LEP and improve their care.

U.S. federal laws outline expectations for the availability of professional translation services for patients within healthcare organizations receiving funds through Medicare and Medicaid programs, but this language accessibility option has not been broadly expanded into digital extensions of healthcare. To ensure equity in access based on language, translations of patient portal elements, including patient registration instructions, log-on sites, and navigation functions should all be translated. Major electronic health record vendors offer translatable options within their patient portal applications, including Epic’s MyChart. The content within the patient portal, including communication with healthcare providers, results of laboratory and imaging, and patient education content, also needs professional translation to ensure language accessibility. In the current state, translation of these materials relies on investment by each healthcare provider group. Continued support through federal and institutional mandates could encourage language accessibility within digital health venues, such as patient portals.

We have several limitations to our study to be mentioned. First, our investigation is cross-sectional in nature, which limits our findings to a brief description at a single point in time for this population. This study is unable to make any statements regarding causality or temporality of the role of LEP among Spanish-preferred individuals, and their ability and willingness to utilize digital health tools. Our study is also limited by differing response rates to our online and mail-based surveys based on age and gender. We deployed the survey in different survey modes to reduce nonresponse bias. We also included formal translation of the survey including the introduction letter and comments by a third-party vendor who specializes in language translation leveraging forward and backward translation. We also pilot-tested our survey instrument in both Spanish and English-speaking individuals and used multiple contact methods including a cross-over design from electronic to paper mail-based invitations to provide several opportunities for as many individuals to respond as possible. Analysis of nonresponse found that compared to responders, nonresponders were younger (median 54.3 years vs. 60.3 years) and had a higher proportion of females. Therefore, our results may be limited in interpretation across these demographic groups. We also had a low response rate overall to our survey request (*n* = 212 completed surveys/2,726 invitations = 7.8%) limiting our ability to generalize our findings to the Spanish-preferred population at large. The response rates we observed are not dissimilar to those achieved by the US government through activities such as the US census (23), and unfortunately we are unable to find any published cross-sectional surveys deployed via paper and/or electronic means to Spanish-preferred individuals that reported response rates. We did deploy three specific tactics to encourage survey complete: complete translation of our survey instruments in the Spanish language by a qualified translation service, expansion of our race categories to include race groups felt to more concordant with respondents based on face validation interviews, and over-sampling of those indicating Spanish is their primary spoken language within our administrative records (23, 24). The majority of our respondents reported ‘Good’ or ‘Very Good’ health, with limited representation of those reporting ‘Poor’ health (6.7%). As those with greater healthcare needs will be those reporting lower self-rated health, our ability to generalize to those populations most seeking healthcare services is limited. Strengths of our study include the use of the Technology Acceptance Model in development of our constructs and inclusion of the digital health literacy assessment tool, which is a validated and published instrument (17).

Improving equitable accessibility to digital tools for individuals with LEP seeking healthcare can help to improve their engagement with their providers and promote self-efficacy in their care. In the current investigation, we identified that Spanish-speaking individuals with LEP experience significant barriers engaging with digital solutions. Opportunities exist with emerging technologies to develop language-concordant healthcare resources that will improve outcomes for this group of patients.

## Data Availability

The raw data supporting the conclusions of this article will be made available by the authors, without undue reservation.
