# Supplementary material for: Digital health literacy and use of patient portals among Spanish-preferred patients in the United States: a cross-sectional assessment
Source: Front Public Health. 2024 Dec 10;12:1455395. doi: 10.3389/fpubh.2024.1455395 (PMC11666482; doi:10.3389/fpubh.2024.1455395)
Supplement: Supplementary file 1 [file Table_1.docx]

| **Supplemental Table.** Assessment for non-response bias among Spanish-preferred speakers by response status. | | | | |
| --- | --- | --- | --- | --- |
|  | Non-Response (N=2514) | Response (N=212) | Total (N=2726) | p value |
| **Age** |  |  |  | <0.0001 |
| N | 2514 | 212 | 2726 |  |
| Mean (SD) | 53.5 (16.9) | 59.5 (14.7) | 54.0 (16.8) |  |
| Median | 54.3 | 60.3 | 55.1 |  |
| Q1, Q3 | 41.0, 65.8 | 50.5, 69.6 | 41.6, 66.3 |  |
| Range | (18.2-99.8) | (20.7-97.3) | (18.2-99.8) |  |
|  |  |  |  |  |
| **Gender** |  |  |  | 0.0368 |
| F | 1606 (63.9%) | 117 (55.2%) | 1723 (63.2%) |  |
| M | 906 (36.0%) | 95 (44.8%) | 1001 (36.7%) |  |
| U | 2 (0.1%) | 0 (0.0%) | 2 (0.1%) |  |
|  |  |  |  |  |
| **Race** |  |  |  | 0.2326 |
| Asian | 38 (1.5%) | 4 (1.9%) | 42 (1.5%) |  |
| Black | 41 (1.6%) | 3 (1.4%) | 44 (1.6%) |  |
| Native American | 10 (0.4%) | 1 (0.5%) | 11 (0.4%) |  |
| Other | 835 (33.2%) | 54 (25.5%) | 889 (32.6%) |  |
| White | 1590 (63.2%) | 150 (70.8%) | 1740 (63.8%) |  |
| (report generated on 23FEB2024) | | | | |
